# Transcriptional regulation of TacL-mediated lipoteichoic acids biosynthesis by ComE during competence impacts pneumococcal transformation

**DOI:** 10.3389/fcimb.2024.1375312

**Published:** 2024-05-08

**Authors:** Miao Yao, Kun Wang, Guangming Song, Yumeng Hu, Jiali Chen, Tingting Li, Longying Liang, Jie Wu, Hongmei Xu, Libin Wang, Yuqiang Zheng, Xuemei Zhang, Yibing Yin, Shifei Yao, Kaifeng Wu

**Affiliations:** ^1^ Department of Laboratory Medicine, The First People’s Hospital of Zunyi (The Third Affiliated Hospital of Zunyi Medical University), Zunyi, Guizhou, China; ^2^ Scientific Research Center, The First People’s Hospital of Zunyi (The Third Affiliated Hospital of Zunyi Medical University), Zunyi, Guizhou, China; ^3^ Key Laboratory of Laboratory Medical Diagnostics Designated by the Ministry of Education, School of Laboratory Medicine, Chongqing Medical University, Chongqing, China

**Keywords:** *Streptococcus pneumoniae*, transformation, competence, teichoic acids, ComE, transcriptional regulation

## Abstract

Competence development is essential for bacterial transformation since it enables bacteria to take up free DNA from the surrounding environment. The regulation of teichoic acid biosynthesis is tightly controlled during pneumococcal competence; however, the mechanism governing this regulation and its impact on transformation remains poorly understood. We demonstrated that a defect in lipoteichoic acid ligase (TacL)-mediated lipoteichoic acids (LTAs) biosynthesis was associated with impaired pneumococcal transformation. Using a fragment of *tacL* regulatory probe as bait in a DNA pulldown assay, we successfully identified several regulatory proteins, including ComE. Electrophoretic mobility shift assays revealed that phosphomimetic ComE, but not wild-type ComE, exhibited specific binding to the probe. DNase I footprinting assays revealed the specific binding sequences encompassing around 30 base pairs located 31 base pairs upstream from the start codon of *tacL*. Expression of *tacL* was found to be upregulated in the Δ*comE* strain, and the addition of exogenous competence-stimulating peptide repressed the *tacL* transcription in the wild-type strain but not the Δ*comE* mutant, indicating that ComE exerted a negative regulatory effect on the transcription of *tacL*. Mutation in the JH2 region of *tacL* upstream regulatory sequence led to increased LTAs abundance and displayed higher transformation efficiency. Collectively, our work identified the regulatory mechanisms that control LTAs biosynthesis during competence and thereby unveiled a repression mechanism underlying pneumococcal transformation.

## 1 Introduction

*Streptococcus pneumoniae* (*S. pneumoniae*) continues to pose a significant global health challenge among children and the elderly, especially in the context of epidemic viral lung infections including SARS-CoV-2 (Li et al., 2023; Zhu et al., 2023). Progress in controlling pneumococcal disease has also been greatly impeded by the emergence of multidrug-resistant isolates and serotype replacement within *S. pneumoniae* strains (Chochua et al., 2017; Yan et al., 2021; Vidal et al., 2022). This issue is closely linked to genetic transformation that is employed by pneumococci for acquiring antibiotic resistance, adapting to their environment, and even generating novel species (Straume et al., 2015; Kim et al., 2019; Lo et al., 2019). Consequently, there has been an increasing interest in investigating the mechanisms underlying gene transfer (Vollmer et al., 2019; Lam et al., 2021; Zhang et al., 2023).

Competence development is crucial for bacterial transformation since it permits the uptake of more efficient exogenous DNA (Claverys et al., 2009; Slager et al., 2019; Lam et al., 2021). In *S. pneumoniae*, this process is triggered by the presence of ComC-derived competence-stimulating peptide (CSP) (Håvarstein et al., 1995). The CSP signal is sensed via the histidine kinase receptor ComD which then undergoes auto-phosphorylation. This process leads to the phosphorylation of its cognate response regulator ComE that is a central component of the adaptive response network. This results in the activation of other network components including the *com*X gene that triggers genes essential for *S. pneumoniae* transformation (competence) (Pestova et al., 1996; Ween et al., 1999). However, it remains to be determined whether there are additional factors regulated by ComE that may also contribute to competence.

Bacterial surface polysaccharides also influence transformation capacity (Weiser and Kapoor, 1999; Li et al., 2019; Minhas et al., 2023). For example, the pneumococcal cell envelope that is primarily composed of capsular polysaccharides (CPS) may impede DNA uptake and hence influence transformation (Weiser and Kapoor, 1999). Non-encapsulated strains demonstrate a higher level of transformability than encapsulated counterparts (Ottolenghi and Macleod, 1963). Teichoic acids (TAs) have also been associated with bacterial transformation, as the transparent phenotype, characterized by elevated TAs and reduced CPS levels and it exhibits enhanced transformation efficiency compared to the opaque phenotype (Weiser et al., 1994; Li et al., 2019; Minhas et al., 2023). Additionally, the deletion of *licD2*, which leads to a reduction of phosphocholine modification in TAs, significantly decreases pneumococcal transformation (Zhang et al., 1999). These findings indicate that cell wall-associated glycopolymers are associated with pneumococcal transformation.

TAs biosynthesis involves multiple operons that collaborate to synthesize the TA precursor chain (Denapaite et al., 2012; Vollmer et al., 2019) that are subsequently linked to either peptidoglycan through LCP phosphotransferase family proteins (Cps2A, Psr and LytR), forming wall teichoic acids (WTAs), or to membrane glycolipids via TacL, forming lipoteichoic acids (LTAs) (Heß et al., 2017; Ye et al., 2018). Interestingly, previous observations have indicated that *tacL* (AE007480) is an *in vivo*-induced gene (Meng et al., 2008). Recently, Minhas et al. revealed that genes involved in TAs biosynthesis are essential and *tacL* mRNA levels decrease during competence (Aprianto et al., 2018; Minhas et al., 2023). These findings suggest that TacL is one of the crucial enzymes that control the TAs ratios in two cellular locations: membrane versus peptidoglycan, which in turn modulates the location of choline-binding proteins, and the regulation of *tacL* during competence may be a key factor that determines transformation. Therefore, its regulation during competence is important to understand.

The *tacL* gene may be transcriptionally regulated during competence so we utilized a *tacL* DNA promoter fragment to physically screen for regulatory proteins capable of binding to the fragment. DNA affinity chromatography was coupled with mass spectrometry (MS) techniques and resulted in the identification of binding proteins, most notably ComE that has previously been established as a transcription factor essential for competence development. Our findings demonstrated that ComE exerts a negative regulatory effect on LTAs biosynthesis by repressing *tacL* transcription. Furthermore, our data indicated that repression of *tacL* by phosphorylated ComE during competence hampers pneumococcal transformation. Overall, these discoveries unveil an innovative mechanism governing pneumococcal transformation through transcriptional regulation of LTAs biosynthesis via phosphorylated ComE.

## 2 Materials and methods

### 2.1 Strains and culture conditions

The bacterial strains, plasmids, and primers utilized in this study are listed in **Supplementary Tables S1, S2**. The *S. pneumoniae* D39 strain (serotype 2, NCTC7466), R6 (non-encapsulated strain, ATCC BAA-255), and their derivatives were cultivated in Todd-Hewitt Broth containing 2% Yeast Extract (THY, Hopebio Qingdao, China) medium or blood agar plates (Autobio, Zhengzhou, China) at 37°C in a 5% CO_2_ atmosphere. *Escherichia coli* (*E. coli*) strains used for cloning and plasmid amplification were grown in Luria-Bertani (LB) broth or on agar plates at 37°C. All bacterial growth media were supplemented with appropriate antibiotics (**Supplementary Table S1**).

### 2.2 Construction of *tacL* mutants and complemented strains

Gene deletion of *tacL* was performed with the long-arm homologous PCR technique (LH-PCR) (Prudhomme et al., 2002; Meng et al., 2008). In brief, we amplified the upstream and downstream regions of *tacL* (GeneBank SPV_1672) from *S. pneumoniae* D39 using primers *tacL* up P1/P2 and *tacL* dw P3/P4, respectively. We also amplified the erythromycin resistance gene from the *S. pneumoniae* CPM8 strain using the *erm*-F/R primer pair. After PCR amplification, these fragments were ligated together by overlapping PCR to create a recombinant fragment. This fragment was then respectively transformed into D39, D39Δ*cps* (SPW3) and R6 strains (The *tacL* gene exhibits high conservation). Colonies were selected on agar plates containing 0.25 μg/mL erythromycin and confirmed through PCR with primers *tacL* up P1 and *tacL* dw P4 to obtain the erythromycin-resistant mutant strain Δ*tacL*. The plasmid pJWV25 (Eberhardt et al., 2009) and the full-length *tacL* fragments were digested with *Spe*I and *Not*I restriction enzymes for cloning. The resulting recombinant plasmids pJWV25-*tacL* (pWKF1) were used to transform into D39Δ*tacL* (SPW1), D39Δ*cps*Δ*tacL* (SPW4) and R6Δ*tacL* (SPW6) strains and were selected on agar plates containing tetracycline to generate the complemented strains D39Δ*tacL::PJWtacL* (SPW2), D39Δ*cps*Δ*tacL::PJWtacL* (SPW5) and R6Δ*tacL::PJWtacL* (SPW7).

### 2.3 Construction of mutants with mutations in the ComE-binding sites of the upstream regulatory sequences of *tacL and cps*

The streptomycin resistance background of strain D39 was utilized for allelic replacement with the counter-selectable Janus cassette (JC) as previously described (Sung et al., 2001). This construct consists of a streptomycin target ribosomal protein S12 and a dominant wild-type *rpsL*+ allele encoding a kanamycin resistance gene. This resulted in the construction of a series of DNA fragments containing ComE*-tacL* promoter binding sequence mutation (Sung et al., 2001; Kirkham et al., 2006). Briefly, a K56T codon change was made in *rspL* to generate the streptomycin-resistant strain D39s (SPW8). Subsequently, upstream (JH up) and downstream (JH dw) sequences of the ComE binding site in *tacL* were amplified using primers JH up-P1/P2 and JH dw-P3/P4 with D39s as a template. The JC sequence was amplified using primers JC-F/JC-R. A recombinant fragment was generated using an overlapping PCR approach with primers JH up-P1/JH dw-P4 (JH up-JC-JH dw) followed by transformation into D39s. Positive recombinant clones D39Δ*JHtacL* (SPW9) selected on kanamycin (200 µg/mL) plates that harbored the JH up-JC-JH dw fragment were identified by PCR using primers JH up-P1/JH dw-P4. D39Δ*JHtacL* indicated that the JH*tacL* sequence was deleted from the genome of the wild-type D39 strain. The binding sequence of ComE-P to *tacL* (JH*tacL*) that exhibited sequence similarity to TATCCTAAATGGT binding sites was identified in the *Streptococcus mutans* genome (Hung et al., 2011). This enabled the construction of mutant sequences for JH1 (complementary paired base sequences), JH2 and JH3 (near complementary paired base sequences) that were then amplified using D39s genomic DNA as a template. Overlapping PCR with primer pairs at both ends yielded recombinant fragments (JH1 up-JH1 dw, JH2 up-JH2 dw, JH3 up-JH3 dw). These recombinant fragments were transformed into D39Δ*JHtacL* followed by plating on agar plates containing streptomycin (150 µg/mL). Positive recombinant clones (D39Δ*JH1* (SPW10), D39Δ*JH2* (SPW 11) and D39Δ*JH3* (SPW 12)) carrying mutations were identified using primers JH1 up-P1/JH1 dw -P4, JH2 up-P1/JH2 dw-P4, JH3 up-P1/JH3 dw-P4.

In a similar manner, we generated D39Δ*JD3* (SPW22) strains using the Janus cassette. In brief, the upstream (JD3 up) and downstream (JD3 dw) were amplified using primers JD3 up-P1/P2 and JD3 dw-P3/P4 with D39 as a template. An overlapping PCR approach with primers JD3 up-P1 and JD3 dw-P4 resulted in a recombinant fragment (JD3 up-JC-JD3 dw) that was then transformed into D39s to generate the D39Δ*JD3.* The *tacL*-up and *tacL*-dw fragments were then amplified by pairs *tacL*-up P1/P2 and *tacL*-dw P3/P4, and this generated the *tacL* deletion fragment using overlapping PCR. These four distinct phenotyping strains for bacterial wall polysaccharides were created. The *tacL* deletion fragment was transformed into the D39Δ*JD3* and D39Δ*cps* as background strains to construct the D39Δ*tacL*Δ*JD3* (SPW23) and D39Δ*tacL*Δ*cps* (SPW24). We similarly transformed the JD3 and CPS (*dexB*-*cps2A*) deletion fragments to D39Δ*JH2* to generate D39Δ*JH2*Δ*JD3* (SPW25) and D39Δ*JH2*Δ*cps* (SPW26).

### 2.4 Construction of *comE* mutants and complemented strains

D39Δ*comE* (SPW13) was generated by homologous recombination. Briefly, the upstream and downstream regions of *comE* were respectively amplified from the D39 strain using *comE* up P1/P2 and *comE* dw P3/P4 primers. Additionally, the erythromycin resistance gene was amplified from *S. pneumoniae* CPM8 using the *erm*-F/R primer pair. These amplicons were ligated by overlapping PCR to create a recombinant fragment that was transformed into D39. Colonies were selected on agar plates containing erythromycin (0.25 μg/mL) and confirmed through PCR with primers *comE* up P1 and *comE* dw P4 to obtain the erythromycin-resistant mutant strain. The mutant strains were complemented using plasmid pJWV25 that contained full-length *comE* and *comE^D58E^* genes. The *comE* ^D58E^ variant was used to mimic the phosphorylated active state of ComE (Martin et al., 2013). The plasmid pJWV25 and DNA fragment were digested by *Spe*I and *Not*I restriction enzymes for subsequent cloning. To obtain the D39::*PJWcomE* ^WT^ (SPW14) and D39::*PJWcomE* ^D58E^ (SPW16) strains, the resulting recombinant plasmids pJWV25-*comE^WT^* (pWKF2) and pJWV25-*comE^D58E^* (pWKF3) were used to transform into the D39 strain and selected on agar plates containing tetracycline (1 μg/mL). and then the *comE* deletion fragment was transformed into the D39::*PJWcomE* ^WT^ and D39::*PJWcomE* ^D58E^ constructed the complemented D39Δ*comE*::*PJWcomE* ^WT^ (SPW15) and D39Δ*comE*::*PJWcomE* ^D58E^ (SPW17) strains.

### 2.5 Construction of luciferase-reporter strains

To construct luciferase reporter strains, the upstream regulatory sequence of *tacL* was amplified from the genomic DNA of the D39 strain using *tacL*-*luc* F/R primer. The plasmid pEVP3-*luc* (pWKF6) and the *tacL* upstream regulatory sequence were digested with *Xho*I-*BamH*I to generate a linearized fragment. The resulting fragment was subsequently inserted into the 3,738-bp long plasmid pEVP3-*luc* that confers chloramphenicol resistance, yielding pEVP3-*PtacL*-*luc* (pWKF7). Similarly, *comE*-*luc* transcriptional fusion was generated in plasmid pEVP3-*luc* by amplifying a DNA fragment of the *comE* promoter from D39 using primer pairs *comE*-*luc* F/R, followed by digestion with *Xho*I/*BamH*I and subsequent ligation to *Xho*I/*BamH*I-digested pEVP3*-luc* to generate pEVP3-*PcomE-luc* (pWKF8). The recombinant plasmid was transformed into D39 to obtain D39::pEVP3-*PcomE* (SPW18) and D39::pEVP3-*PtacL* (SPW20) strains. Subsequently, the *comE* deletion fragment was transformed into the D39::pEVP3-*PcomE* and D39::pEVP3-*PtacL* to generate D39Δ*comE::*pEVP3*-PcomE* (SPW19) and D39Δ*comE::*pEVP3*-PtacL* (SPW21) strains.

### 2.6 DNA affinity chromatography-pulldown

D39 cell lysates were collected in cell cultures at OD_595nm_ of 0.5 and subjected to repetitive freeze-thawing followed by ultrasonic fragmentation to obtain cell lysates (200~300 µg/mL) (Jutras et al., 2012). A 207-bp DNA fragment (400 µg/mL) was labeled with biotin at 5’-end and amplified from the D39 genomic DNA strain using Pulldown-*tacL* F/R primers. Subsequently, the purified target and non-specific DNA probe were immobilized separately on streptavidin-coated M-280 immunomagnetic beads and co-incubated with D39 cell lysates. The mixture was subjected to purification using elution buffers containing varying NaCl concentrations. The eluted proteins were separated by SDS-PAGE and stained with Coomassie blue, and compared to the control group for enrichment analysis. Finally, the enriched proteins were excised from the stained gels and identified by mass spectrometry.

### 2.7 Recombinant protein preparation

The pneumococcal *comE* and *comE* ^D58E^ gene from *S. pneumoniae* D39 was amplified by PCR, sequenced to ensure accuracy. The pET-28α (+) plasmid and *comE* ^D58E^/*comE* ^WT^ fragments were subjected to restriction enzyme digestion and subsequent ligation to generate recombinant plasmids pET-28a-*comE^WT^* (pWKF4) and pET-28a-*comE^D58E^* (pWKF5). Subsequently, they were transformed into *E. coli* BL21 (DE3) and protein production were induced by the addition of isopropyl-β-d-thiogalactoside (IPTG) to 1 mM. Following incubation, the cultures were collected and resuspended in PBS prior to sonication. The recombinant protein was purified from the cell lysate using Nickel column affinity chromatography and stored at -80°C.

### 2.8 Electrophoretic mobility shift assay

The protein-DNA binding assays were performed using the Scientific Light-Shift kit (Thermo Fisher, Pittsburg, PA, USA) according to the manufacturer’s protocol. The purified ComE^D58E^ and ComE^WT^ proteins were respectively incubated in a reaction buffer (1 × binding buffer, 2.5% glycerol, 5 mM MgCl_2_, 5 mM ZnCl_2_, 50 ng/μL Poly (dI-dC), 0.05% NP-40) at 4°C for 5 min, followed by addition of biotin-labelled or un-labelled DNA probes and incubation at 37°C for 30 min. Samples were electro-transferred onto nylon membranes (Bio-Rad, Hercules, CA, USA) in a solution containing 0.5 × Tris-borate-EDTA (TBE) and subjected to UV crosslinking. Finally, band images were captured using a chemiluminescent imaging system (Bio-Rad).

### 2.9 DNase I footprinting assay

The 400 ng probes for *tacL* (P*tacL*) were incubated with varying amounts of ComE^D58E^ in a total volume of 40 µL to incubate for 30 min at 25°C. DNase I (Promega, Madison, WI, USA) was further added to the reaction system and incubated for 1 min at 37°C. Subsequently, the samples underwent phenol-chloroform extraction and ethanol precipitation and the resulting DNA was dissolved in 30 µL Mini-Q water. The prepared DNA was analyzed using an automated DNA analyzer (ABI Prism 3100 DNA sequencer; Thermo Fisher).

### 2.10 Western blot and dot-blot assays

Bacterial suspensions were collected at 4°C that were washed with PBS and resuspended in PBS to an optical density OD_595nm_ of 0.5 to normalize the sample. Lysis buffer containing 0.5% deoxycholate (Sigma) was then added and the solutions were incubated at 37°C for 15 min. Samples were boiled for 10 min, subjected to SDS-PAGE on a 12% gel, and electro-transferred onto Immunobilon (Millipore, Burlington, MA, USA) and incubated for 2 h at room temperature in Tris-buffered saline with 0.1% Tween 20 (TBST) solution containing 5% skim milk. The membranes were incubated overnight at 4°C with the primary antibody and then washed three times with TBST. The primary antibodies used included rabbit anti-CWPS (Identification of TAs, 10-25kDa defined as LTAs (Wu et al., 2014; Heß et al., 2017; Flores-Kim et al., 2019)) IgG (1: 1000) which targeted TAs as well as mouse anti-ComE (1: 1000). A peroxidase-conjugated secondary antibody goat anti-rabbit IgG antibody was utilized 1: 8000 and the blot was developed using a Clarity Western Enhanced chemiluminescence (ECL) substrate kit (Epizyme, Cambridge, MA, USA) and visualized on a gel imager. Dot blot analysis utilized transfer membranes activated in methanol for 1 min and subsequently washed in PBS to achieve charge equilibrium. Bacterial lysates (2.5 μL) were serially diluted to the indicated concentrations before being applied to the activated transfer membrane and allowed to fully dry. Subsequent steps followed the protocols described in Western blot.

### 2.11 Immunosorbent assays (ELISA)

Bacteria were cultured in THY until OD_595nm_ of 0.5 and harvested at 4°C, then was resuspended in PBS. The bacterial suspension was diluted with the antigen coating solution and spread onto 96-well plates overnight at 4°C and then rinsed three times with PBS followed by blocking in PBS containing 2% BSA for 1 h at 37°C. The primary antibody rabbit anti-CWPS (1: 1000) and secondary antibody goat anti-rabbit IgG (1: 8000) were used for processing. After color development, the TAs content was measured by absorption at 450 nm.

### 2.12 Fluorescence activated cell sorting

*S. pneumoniae* cultured in THY medium were harvested at 10000 × g for 3 min upon reaching an OD_595nm_ of 0.5 and washed three times with PBS containing 0.05% Tween 20. The resulting pellets were resuspended in 100 µL 1% BSA in PBS and incubated with (rabbit anti-CWPS) 1: 50 for 1 h at 37°C. Subsequently, the pellets were washed as per above and stained with phycoerytherin (PE)-labeled goat anti-rabbit IgG for 1 h at 37°C. The samples were suspended in 200 µL PBS and analyzed using a BD FACS Calibur flow cytometer (BD Biosciences, Franklin Lakes, NJ, USA).

### 2.13 Real-time PCR

Bacteria were harvested by centrifugation at 10000 × g for 3 min at 4°C and the cell pellets were resuspended in lysis buffer containing 0.5% deoxycholate (Sigma) and incubated at 37°C. The total RNA was isolated from strains using the Trizol reagent (Takara, Shiga, Japan) and RNA (1 μg) was reverse transcribed to cDNA using an iScript gDNA synthesis kit (Takara). The mRNA levels of the target genes were quantified using real-time PCR using Power SYBR Green PCR Master Mix (TsingKe Biotech, Beijing, China), following the manufacturer’s protocol for relative quantitative analysis. The primers used for PCR are listed in **Supplementary Table S2**. The *gyrB* served as internal control.

### 2.14 Luciferase assays

Bacteria containing luciferase reporter strains were cultivated in the competence medium (THY containing 20 × BSA+CaCl_2_) until OD_595nm_ of 0.1. Exogenous transformation inducer (CSP, 100 µg/mL) and D-luciferin (Lablead) at a concentration of 2.7 mg/mL were added as previously described (Liu et al., 2017). Luminescence assays were performed in Polystyrol 96-well plates (Corning) at 37°C. The absorbance at OD_595nm_ and luminescence measured as relative luminescence units (RLU) were determined for each strain. The optical density measurements at OD_595nm_ were taken every 5 min and used to normalize luciferase activity.

### 2.15 Transformation assays

The pneumococcal strains were cultured in the competence medium supplemented with 20 × BSA + CaCl_2_ until OD_595nm_ of 0.1. CSP (EMRLSKFFRDFILQRKK) was then added to 100 µg/mL and the mixture was incubated for 10 min at 37°C. The *E. coli*-*S. pneumoniae* shuttle vector pIB166 (Biswas et al., 2008) was incubated for 30 min on ice followed by incubation for 90 min at 37°C. The resulting samples were plated on blood agar plates in the presence and absence of 25 μg/mL chloramphenicol to enumerate total numbers and positive transformants. The transformation efficiency was calculated as the ratio between the number of antibiotic-resistant colony-forming units (CFUs) and the number of CFUs on nonselective blood agar plates.

### 2.16 Statistical analysis

Statistical significance between groups was compared using variance analysis *ANOVA* or Student’s *t*-test. All statistical analyses were performed using GraphPad Prism version 8.00 (San Diego, CA, USA). Data are presented as means ± SD. Statistical significance was defined as a *P*-value less than 0.05.

## 3 Results

### 3.1 Deletion of *tacL* impairs pneumococcal transformation

**Figure 1:**
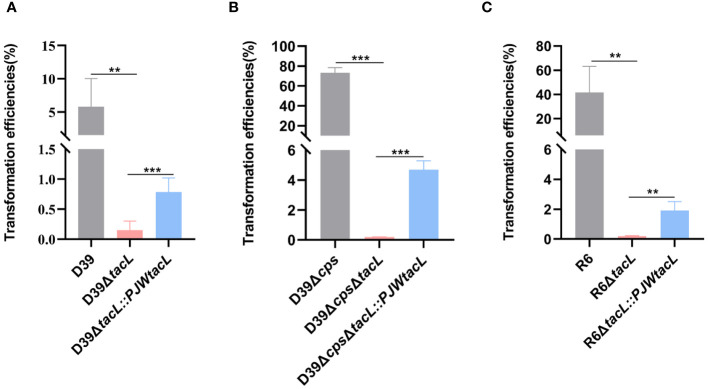
Deletion of *tacL* impairs pneumococcal transformation. **(A)** Transformation efficiency of wild-type D39, D39Δ*tacL* and the *tacL* complemented strains. **(B)** Transformation efficiency of non-encapsulated *S. pneumoniae* D39Δ*cps*, D39Δ*cps*Δ*tacL* and complemented strains. **(C)** Transformation efficiency of non-encapsulated *S. pneumoniae* R6, R6Δ*tacL* and complemented strains. Statistical analysis was performed using an unpaired two-tailed Student’s *t*-test with significance levels denoted as ***P < 0.001, **P < 0.01.

To investigate the impact of *tacL* on pneumococcal transformation, we generated a *tacL* deletion mutant using an erythromycin for allelic replacement. The complete *tacL* sequence was cloned into plasmid pJWV25 to construct a complemented strain with *tacL* (D39Δ*tacL*::*PJWtacL*). To eliminate the potential impact of integration processes on exogenous DNA uptake and accurately reflect the influence of competence on transformation, we employed the non-integrating plasmid pIB166 as the donor. We found a significant impairment in the transformation efficiency of D39Δ*tacL* compared to wild-type D39. The complemented strain exhibited an increased transformation efficiency that did not reach the level observed in the wild-type strain but was still significantly higher than the deletion of *tacL* strain (**Figure 1A**).

These transformation assays were then conducted using non-encapsulated strains to eliminate any potential interference caused by CPS that is known to affect transformation efficiency. In the non-encapsulated strain D39Δ*cps*, the *tacL* deletion resulted in a significant decrease in transformation efficiency compared to the cognate parental strain and the complemented strain exhibited higher transformation efficiency than the *tacL* deletion strain (**Figure 1B**). Similarly, the deletion of *tacL* resulted in compromised transformation efficiency in the R6 (non-encapsulated) strain background (**Figure 1C**). These findings underscore the importance of *tacL* in pneumococcal transformation.

### 3.2 Identification of potential regulators capable of binding to the upstream regulatory fragment of *tacL*

**Figure 2:**
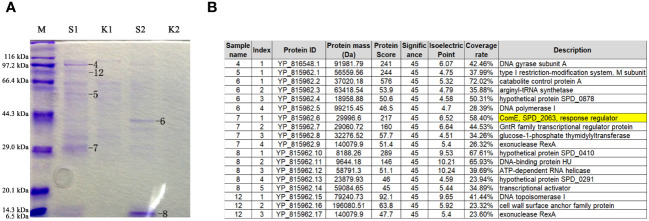
Identification of regulatory proteins capable of binding to the fragment of governing *tacL* expression. **(A)** Enriched proteins subjected to SDS-PAGE gel analysis. lane M: Protein molecular mass standards. S1 and S2: elution fractions obtained by binding with the purified specific target probe using 100 mM and 200 mM NaCl, respectively, the numbers 4-8 and 12 represent gel-excised and used for MS analysis. K1 and K2: elution fractions obtained by binding with the non-specific probe using 100 mM and 200 mM NaCl, respectively. **(B)** Proteins identified by mass spectrometry.

To establish a correlation between *tacL* transcription and transformation, we performed DNA pull-down assays coupled with mass spectrometry (MS) analyses. A 207-bp long regulatory DNA probe of *tacL* was labeled at the 5’-end with biotin. The probe was then incubated with pneumococcal lysates and subjected to DNA affinity chromatography-pulldown. The binding proteins were separated by SDS-PAGE (**Figure 2A**) and subsequently subjected to MS analysis and this led to the identification of several potential regulatory proteins that included ComE, GntR family SPD_0064, MarR family SPD_0379, GlnR, and RitR25 (**Figure 2B**). Notably, ComE is a well-established protein that plays a pivotal role in *S. pneumoniae* transformation (Martin et al., 2010).

### 3.3 The phosphomimic ComE specifically binds to the upstream regulatory sequences of *tacL*

**Figure 3:**
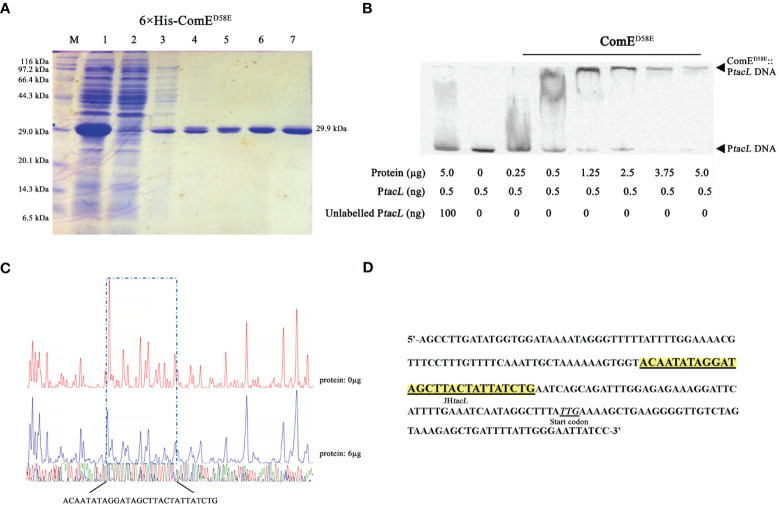
Phosphomimic ComE^D58E^ binds to the upstream regulatory region of *tacL*. **(A)** The recombinant 6×His-ComE^D58E^ protein was analyzed by SDS-PAGE and migrated with an apparent molecular mass of 29.9 kDa, Lane M: Marker; Lane 1, Protein expression induced by IPTG; Lane 2: Ni-NTA flow-through; Lanes 3 – 6: elution from the columns with imidazole at 20, 40, 80 and 500 mM, respectively. Lane 7: the purified form of 6×His-ComE^D58E^. **(B)** EMSA results of binding between ComE^D58E^ and P*tacL* as indicated. **(C)** Sequence analysis of DNase I footprinting protection assay between ComE^D58E^ and the *tacL* DNA probe. **(D)** Structural organization of the *tacL* promoter-proximal region. The start codon was indicated. Binding sequences for ComE^D58E^ are highlighted in yellow.

The transcription factor ComE is essential for competence development in *S. pneumoniae* (Ween et al., 1999; Martin et al., 2010), undergoes phosphorylation at aspartic acid residue 58 (D58) during competence, thereby triggering the expression of early competence genes (Martin et al., 2013). EMSA assays were conducted to validate the binding between ComE or its phosphomimic form and the DNA probe. Distinct shift bands were observed in a dose-dependent manner upon increasing concentrations of ComE^D58E^ (**Figures 3A, B**) while no shift bands were detected for ComE^WT^ (**Supplementary Figure S1**). DNase I footprinting revealed the presence of a protected region approximately 30 base-pairs in length (**Figure 3C**) that was identified 31 base-pairs upstream from the start codon (**Figure 3D**). These findings indicated that phosphomimic ComE^D58E^ exhibited the specific binding affinity towards the upstream regulatory sequence of *tacL*.

### 3.4 Mutation of the JH*tacL* alters the transcription of *tacL* and LTAs levels

**Figure 4:**
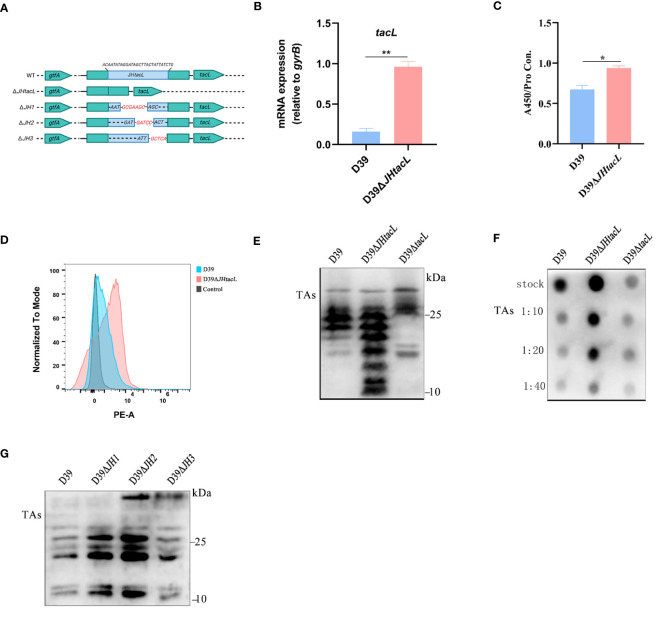
Deletion of JH*tacL* resulted in increased transcription of *tacL* and levels of LTAs. **(A)** Mutation patterns of the binding sites of JH*tacL*. **(B)** The transcript levels of *tacL* for the indicated strains. **(C)** ELISA analysis for TAs detection. **(D)** Flow cytometry to determine the surface exposed TAs. **(E)** Western blot and **(F)** Dot blot analysis of TAs for the indicated strains. **(G)** Western blot analysis of TAs of wild-type D39 and the indicated *tacL* promoter binding sequence mutations for a series of mutants (*JH1/JH2/JH3*). Bacteria were harvested at OD_595nm_ of 0.5 for *tacL* transcription and TAs analysis, we used an bacterial density (defined as an OD_595nm_ of 0.5) for normalizing. Statistical analysis was carried out using unpaired two-tailed Student’s *t*-test. ** P < 0.01 and * P < 0.05.

The impact of the regulatory sequence of *tacL* on LTAs biosynthesis was demonstrated by generating a D39Δ*JHtacL* strain, in which the binding region identified by DNase I footprinting assay was deleted (**Figure 4A**). The mRNA expression level of *tacL* in D39Δ*JHtacL* was significantly upregulated compared to that in the wild-type strain (**Figure 4B**). ELISA, flow cytometry and dot-blot analyses revealed an increased abundance of teichoic acids and an up-regulated LTAs (manifested as smaller molecular-weight bands 10 and 25 kDa) was displayed by western blot in the *JHtacL* mutant when compared with the wild-type strain (**Figures 4C–F**).

To determine the contribution of the regions within JH*tacL* that associate with phosphorylated ComE, a series of mutants were constructed to evaluate LTAs biosynthesis (**Figure 4A**). Among all tested site mutants, the *JH2* mutant exhibited significantly enhanced LTAs production (**Figure 4G**). Collectively, these findings provide compelling evidence that the JH*tacL* region is involved in the regulation of *tacL* transcription, thereby exerting an influence on the biosynthesis of LTAs.

### 3.5 ComE negatively regulates *tacL* transcription during competence

**Figure 5:**
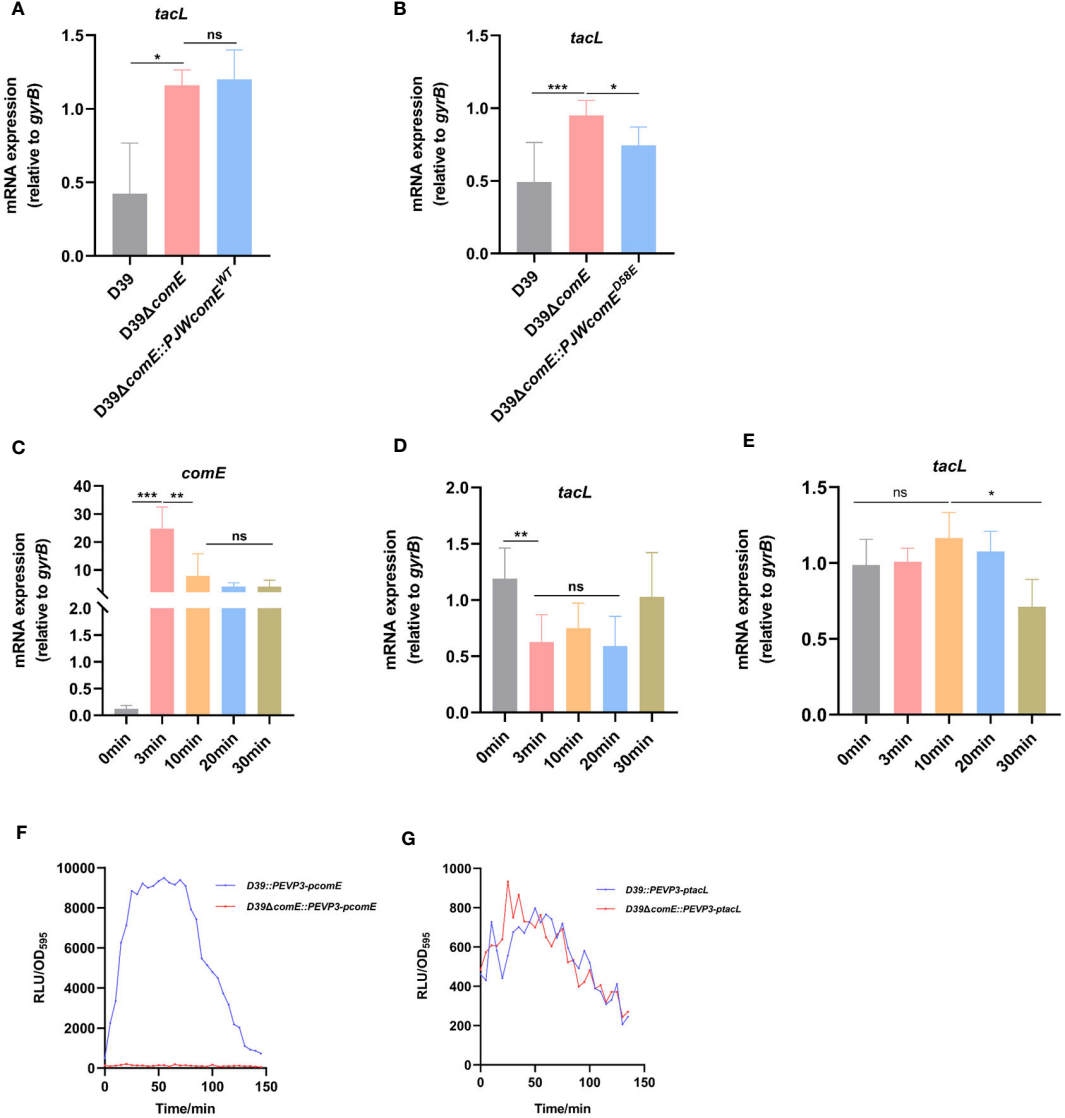
ComE negatively regulates *tacL* transcription during competence. **(A, B)** RT-PCR analysis of *tacL* expression in **(A)** the wild-type D39, D39Δ*comE*, D39Δ*comE::PJWcomE^WT^* and **(B)** D39Δ*comE:: PJWcomE^D58E^*. Bacteria were harvested at OD_595nm_ of 0.5. The mRNA expression levels of *tacL* were normalized to *gyrB* expression. **(C–E)** RT-PCR to determine expression levels of **(C)** *comE* and **(D)** *tacL* in the wild-type D39 and examined the transcription of **(E)** *tacL* in D39Δ*comE*. These strains were grown in competence THY medium until OD_595nm_ 0.1 and induced with CSP for different durations (0 min, 3 min, 10 min, 20 min and 30 min). The mRNA expression levels of *tacL* were normalized to *gyrB* expression. **(F, G)** Transcriptional activity of **(F)** *comE* and **(G)** *tacL* was assessed using luciferase assays for the wild-type D39 and D39Δ*comE* (*comE*-*luc*, *tacL*-*luc*) strains. These strains were grown to OD_595nm_ 0.1 followed by the addition of 100 µg/mL of CSP and 2.7 mg/ml of luciferase. Luciferase activity and OD_595nm_ were measured at 5 min intervals. Statistical analysis was performed using the unpaired two-tailed Student’s *t*-test or one-way *ANOVA* with Tukey’s multiple-comparison test. Statistical significance was denoted as *** P < 0.001, ** P < 0.01, * P < 0.05, while ns indicated non-significance.

We postulated that ComE may exert regulatory control over the transcription of *tacL* during competence. By generating the D39Δ*comE* mutant and its complementary strain, we observed a significant increase in mRNA levels of *tacL* in the *comE* mutant compared to the wild-type D39 strain, while no significant changes were observed in D39Δ*comE*::*PJWcomE* ^WT^ (no available antibody for TacL, we resorted to assessing the transcript level of *tacL*) (**Figure 5A**). The active form of ComE protein, as assessed using a phosphomimic ComE^D58E^, was previously identified as a regulator of *cps* locus transcription (Zheng et al., 2017). While, the ComE may be not activated to ComE^D58E^ during the bacteria’s logarithmic growth phase. Therefore, we constructed a complemented strain expressing ComE^D58E^ (Δ*comE*::*PJWcomE* ^D58E^) to evaluate its impact on *tacL* transcription. We found a decreasing trend was evident in the complementing strain D39Δ*comE*::*PJWcomE* ^D58E^ (**Figure 5B**). These findings indicated that phosphorylated ComE is the form of the protein required to repress *tacL* transcription.

To further elucidate this regulatory mechanism, we induced competence in the D39 strain by exogenous supplementation with CSP and monitored the expression of *comE* and *tacL*. During the initial phase, there was an upregulation in *comE* mRNA level (**Figure 5C**) while *tacL* exhibited a transient downregulation in the wild-type strain (**Figure 5D**), consistent with the transcriptome data was reported (Aprianto et al., 2018). The transcription of *tacL* remained unaltered following CSP induction in the D39Δ*comE* mutant (**Figure 5E**). The luciferin reporter assay demonstrated enhanced transcriptional activity of *comE* (**Figure 5F**) while *tacL* experienced a transient decrease in D39 but remained unchanged in the D39Δ*comE* strain upon CSP stimulation (**Figure 5G**). These findings indicated that ComE exerts a negative regulatory effect on *tacL* transcription during the initial phase of competence.

### 3.6 ComE restricts LTAs biosynthesis during competence

**Figure 6:**
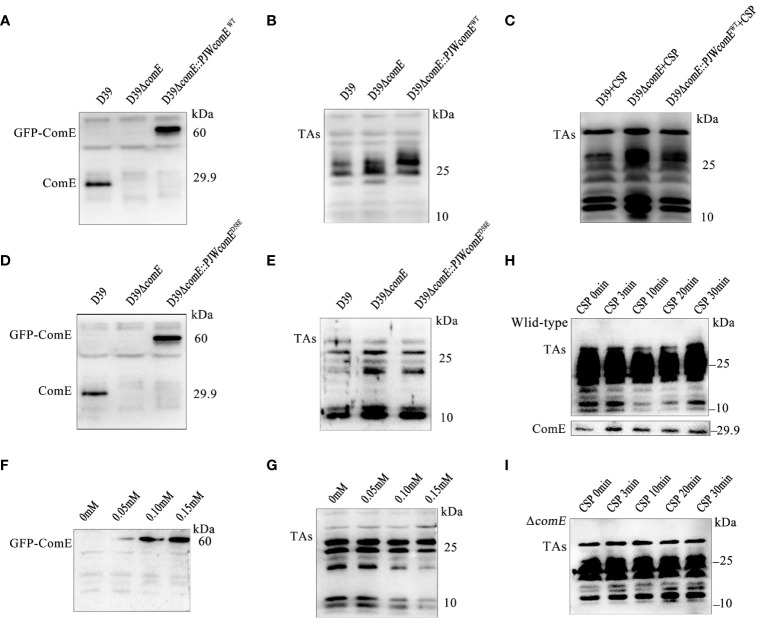
ComE negatively regulates LTAs production. **(A, B)** Western blot analysis levels of **(A)** ComE and **(B)** TAs in the indicated strains (wild type D39, D39Δ*comE* and D39Δ*comE::PJWcomE^WT^*), cultured to OD_595nm_ of 0.5. **(C)** Western blot analysis of TAs levels for the indicated strains (wild type D39, D39Δ*comE* and D39Δ*comE::PJWcomE^WT^*). These strains cultured to OD_595nm_ 0.1 followed by CSP induction for 15 min. Bacterial suspensions were harvested and adjusted for bacterial density (defined as an OD_595nm_ of 0.5) for normalization. **(D, E)** Western blot analysis to determine levels of **(D)** ComE and analyze **(E)** TAs for the wild-type D39, D39Δ*comE*, and D39Δ*comE:: PJWcomE^D58E^* strains, cultured to OD_595nm_ of 0.5. **(F, G)** Western blot to determine levels of **(F)** ComE and **(G)** TAs for the D39Δ*comE*:: *PJWcomE^D58E^* strain in the presence of differing levels of ZnCl_2_ in the culture medium (0 mM, 0.05 mM, 0.10 mM and 0.15 mM) cultured to OD_595nm_ of 0.5. **(H, I)** Western blot to assess the levels of TAs and ComE for the **(H)** wild-type D39 and **(I)** D39Δ*comE*. The strains were cultured to OD_595nm_ 0.1 followed by CSP induction for different durations (0 min, 3 min, 10 min, 20 min and 30 min). Bacterial suspensions were harvested and adjusted for bacterial density (defined as an OD_595nm_ of 0.5) for normalization.

To demonstrate the regulatory role of ComE in TAs biosynthesis, we constructed the strains D39Δ*comE* and D39Δ*comE::PJWcomE* ^WT^. The expression of ComE was absent in D39Δ*comE* while GFP-ComE could be detected in the complemented strain (**Figure 6A**). The *comE* mutant displayed significantly elevated levels of LTAs (manifested as smaller molecular-weight bands 10 and 25 kDa) while the overall level of LTAs in the complemented strain remained unchanged compared to that in the Δ*comE* mutant (**Figure 6B**). *S. pneumoniae* senses the CSP signal, the histidine kinase receptor ComD that undergoes autophosphorylation causing its cognate response regulator ComE to phosphorylate (Pestova et al., 1996; Martin et al., 2013). Therefore, exogenous CSP was used to induce the phosphorylation of ComE. We found that the complemented strain (D39Δ*comE::PJWcomE^WT^*) induced by CSP displayed inhibition of LTAs content (**Figure 6C**). Meanwhile, the complemented strain expressing ComE^D58E^ (Δ*comE::PJWcomE^D58E^*) also exhibited a significant reduction in LTAs synthesis in **Figures 6D, E**. These findings suggested that phosphorylated ComE is involved in regulating the LTAs biosynthesis.

To validate the effect of phosphorylated ComE on LTAs biosynthesis, the D39Δ*comE*-*PJWcomE* ^D58E^ strain was grown in THY medium supplemented with varying concentrations of ZnCl_2_ to fine-tune the expression of ComE^D58E^. ZnCl_2_ exposure led to a concentration-dependent up-regulation of GFP-ComE^D58E^ expression (**Figure 6F**) resulting in decreased levels of LTAs (**Figure 6G**). While a clear suppression of LTAs biosynthesis during competence was evident for the wild-type D39 (**Figure 6H**), no corresponding inhibitory effects were observed in D39Δ*comE* during competence (**Figure 6I**). These findings strongly indicated that phosphorylated ComE plays a crucial role in inhibiting LTAs biosynthesis during competence.

### 3.7 Abolished regulation of LTAs and CPS by ComE affects pneumococcal transformation

**Figure 7:**
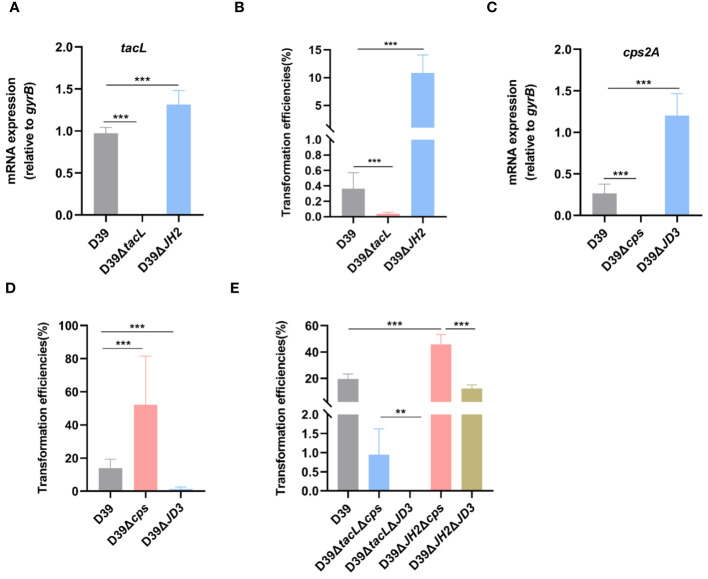
TacL-mediated LTAs biosynthesis affects pneumococcal transformation. **(A)** RT-PCR analysis of mRNA levels of *tacL* for the indicated strains (wild-type D39, D39Δ*tacL* and D39Δ*JH2*) harvested at OD_595nm_ 0.5. The mRNA levels were expressed relative to *gyrB.* **(B)** Transformation efficiency of the indicated strains (wild-type D39, D39Δ*tacL* and D39Δ*JH2*) that expressed varying LTAs levels. **(C)** RT-PCR analysis of mRNA levels of *cps2A* for the indicated strains (wild-type D39, D39Δ*cps* and D39Δ*JD3*). **(D)** Transformation efficiency of the indicated strains (wild-type D39, D39Δ*cps* and D39Δ*JD3*) that expressed varying CPS contents. **(E)** Transformation efficiency of different bacterial phenotypes as indicated (D39, Δ*tacL*Δ*cps*, Δ*tacL*Δ*JD3*, Δ*JH2*Δ*cps* and Δ*JH2*Δ*JD3*). Statistical analysis was performed using unpaired two-tailed Student’s *t*-test or one-way *ANOVA* with Tukey’s multiple-comparison test. Significance differences were denoted as *** P < 0.001; ** P < 0.01.

To investigate the impact of ComE-regulated LTAs biosynthesis on pneumococcal transformation, we compared the *tacL* transcript levels and transformation efficiency among wild-type D39, D39Δ*JH2*, and D39Δ*tacL* strains. The *tacL* mRNA could not be detected in D39Δ*tacL* strain. In contrast to the wild-type strain, elevated levels of *tacL* transcript were observed in D39Δ*JH2* (**Figure 7A**) and this correlated with a significantly enhanced transformation efficiency (**Figure 7B**).

A previous study reported that ComE functions as a transcriptional repressor of CPS biosynthesis and thereby exerts a negative regulatory effect via binding to the promoter region JD3 of the *cps* gene (Zheng et al., 2017). We therefore confirmed that the D39Δ*JD3* strain exhibited high expression levels of capsular polysaccharides (**Supplementary Figure S2**). We further validated the impact of ComE-regulated CPS biosynthesis on pneumococcal transformation and evaluated the transformation efficiency of the JD3 mutant. In comparison to the wild-type D39, D39Δ*cps* exhibited a significant enhancement in transformation efficiency, whereas it was suppressed in the JD3 mutant (**Figures 7C, D**).

Furthermore, transformation efficiencies of mutants generated by combining the ComE-binding sites of *tacL* with *cps* regulatory sequences revealed that strains with higher LTAs levels (Δ*JH2*Δ*cps* and Δ*JH2*Δ*JD3*) exhibited significantly enhanced transformation abilities compared to the other strains with lower levels in Δ*tacL*Δ*cps* and Δ*tacL*Δ*JD3*. Additionally, the pneumococcal transformation of the strains with higher CPS (Δ*tacL*Δ*JD*3 and Δ*JH*2Δ*JD*3) was found to be suppressed in comparison to the D39Δ*tacL*Δ*cps* and D39Δ*JH*2Δ*cps* (**Figure 7E**). These findings indicated that ComE regulates both CPS and LTAs biosynthesis and this subsequently influences pneumococcal transformation capacity.

## 4 Discussion

This study provides novel and significant insights into the regulation of teichoic acids biosynthesis during pneumococcal competence. Our findings demonstrate that phosphomimic ComE specifically binds to regulatory sequences spanning approximately 30 base pairs located 31 base pairs upstream from the start codon of the *tacL* gene, resulting in repression of *tacL* transcription. Moreover, our results emphasize the importance of regulation of TAs biosynthesis during competence and establish the involvement of *tacL*-mediated LTAs biosynthesis in pneumococcal transformation.

A previous CRISPRi-seq analysis indicated that *tacL* expression is suppressed during competence (Aprianto et al., 2018). We sought to identify the precise mechanism that underlies this inhibition. We screened the potential transcriptional regulators involved in linking competence with *tacL* and identified several potential regulators, including ComE. In *S. pneumoniae*, ComE undergoes phosphorylation in the presence of phosphorylated ComD, a transmembrane protein histidine kinase responsible for sensing CSP (Yang and Tal-Gan, 2019). Phosphorylated ComE is a central component of the cellular adaptive-response network, demonstrating a pivotal role in gene regulation, including *comX* gene that triggers genes essential for *S. pneumoniae* transformation (Kowalko and Sebert, 2008; Martin et al., 2013). Given its well-established function as a transcription factor and its involvement in competence (Martin et al., 2010; Zheng et al., 2017), we focused on elucidating the transcriptional regulatory mechanism used by ComE to regulate *tacL* expression. Since ComE phosphorylation at D58 is associated with competence, we expressed a recombinant functional phosphomimic ComE^D58E^ and conducted EMSA assays to unveil their interaction. We observed that ComE^D58E^ specifically bound to the *tacL* regulatory region. *In vitro* footprinting experiments also indicated that ComE^D58E^ bound to the regulatory region of *tacL*, and there was a specific binding sequence. We also observed that LTAs expression patterns were altered in the strain following deletion of the JH*tacL* sequence from the *tacL* promoter. This phenomenon may be caused by unpolymerized RU of TAs or other factors that we did not identify. Moreover, the JH*tacL* sequence exhibits sequence similarity with the TATCCTAAATGGT binding sites identified in *S. mutans* (Hung et al., 2011). The transcription of *tacL* is repressed by ComE during competence, resulting in the inhibition of LTAs biosynthesis. The repression is primarily attributed to the binding of ComE to the upstream regulatory region of *tacL*. Notably, CSP stimulation no longer reduces LTAs biosynthesis in *comE* mutants. Collectively, these findings indicate that ComE regulates the transcription of *tacL* by binding to its upstream regulatory region, thereby linking competence and TAs remodeling.

Upon CSP stimulation, we also found an immediate up-regulation of ComE protein levels, resulting in a reduction in *tacL* transcription (**Figure 5D**). As the response progressed, *comE* mRNA levels remained up-regulated and the *tacL* transcripts increased (**Figures 5C, D**). These findings suggested the presence of a complex regulatory network governing *tacL* transcription.

The *tacL* gene deletion strongly reduced CSP-induced transformation and agreed with a previous observation emphasizing the importance of phosphocholine modifications in TAs for pneumococcal transformation (Zhang et al., 1999; Minhas et al., 2023). Therefore, we hypothesized that *tacL* might be upregulated during competence. However, our findings indicated the contrary; the phosphomimic ComE suppressed the transcription of *tacL* during competence, and mutation in the binding sites resulted in increased *tacL* levels and LTAs amounts that promoted transformation. These results suggested a complex role of *tacL*-mediated LTAs biosynthesis during competence that requires further clarification.

Bacterial cell wall polysaccharides play numerous roles in transformation (Mirouze et al., 2018; Minhas et al., 2023) and in particular, CPS impedes pneumococcal transformation (Weiser and Kapoor, 1999; Li et al., 2019). Furthermore, phosphorylated ComE exerts a repressive effect on *cps* expression by binding to the *cps* promoter region, thereby inhibiting CPS biosynthesis (Zheng et al., 2017). However, it remains unclear whether this regulatory mechanism contributes to the process of pneumococcal transformation. Our findings underscore the significant involvement of ComE-mediated *cps* transcription in pneumococcal transformation, as evidenced by the impaired transformation resulting from mutations in the regulatory sequences of the *cps* locus This also partially reveals why competent bacteria possess lower levels of CPS (Weiser and Kapoor, 1999; Minhas et al., 2023). Collectively, we propose that cell wall remodeling is an event driven by ComE at the transcriptional level.

The competence response has been demonstrated to potentially induce fatal bacterial injury (Claverys et al., 2007; Straume et al., 2015). Therefore, stringent regulation of competence is imperative in order to prevent bystander damage. LTAs are glycopolymers that play crucial roles in cell division, surface adhesion and biofilm formation (Moscoso et al., 2006; Heß et al., 2017; Ye et al., 2018). The precise reasons underlying the simultaneous production of both LTAs and WTAs by *S. pneumoniae* remain incompletely understood. Activation of choline-binding proteins on the bacterial wall occurs during competence, resulting in lysis of non-competent pneumococci (Eldholm et al., 2010; Wei and Håvarstein, 2012). Simultaneously, immunity proteins within the competent pneumococcal cell walls are activated to provide protection against cleavage (Håvarstein et al., 2006; Straume et al., 2017). *S. pneumoniae* exhibits a phenomenon known as “fratricide” where competent cells engage in natural genetic transformation lyse and thereby eliminate noncompetent siblings present within their environment (Claverys et al., 2007). This phenomenon relies on the activation of the late competence protein CbpD. Competent pneumococci are protected from fratricide by producing the immunity proteins ComM and LytR that are regulated by the transcription factor ComE (Eldholm et al., 2009; Straume et al., 2015; Minhas et al., 2023). This subsequently results in an elevation of surface WTAs levels that function as a protective mechanism against lysis (Minhas et al., 2023). Although we did not provide evidence demonstrating the influence of ComE-regulated LTAs biosynthesis on autolysin activity, based on previous findings (Flores-Kim et al., 2019; Minhas et al., 2023), it is plausible to propose that the involvement of *tacL*-mediated LTAs biosynthesis regulated by ComE in competence is accountable for the regulation of cell wall lysis. The ComE-*tacL*-LTAs pathway may represent a novel molecular mechanism contributing to pneumococcal transformation. The regulation of fratricide activity partially elucidates the necessity for *S. pneumoniae* to produce both LTAs and WTAs.

In conclusion, our study provides compelling evidence that transcriptional repression of *tacL*-mediated LTAs biosynthesis by ComE constitutes a pivotal mechanism underlying the remodeling of the cell wall during competence, thereby exerting an impact on pneumococcal transformation.

## Data availability statement

The original contributions presented in the study are included in the article/**Supplementary Material**, further inquiries can be directed to the corresponding author/s.

## Author contributions

KFW, SY, MY and KW designed experiments. MY, GS, YH, JC, TL and LL carried out experiments. KW, JW, YZ, LW and HX analyzed experimental results. MY, KW and GS wrote the manuscript. KFW, SY, MY, YY and XZ reviewed and edited the manuscript. KFW, SY, and MY: Writing – review & editing and Writing – original draft. GS: Writing – original draft. KW, YH, JC, TL, LL, JW, HX, LW, YZ, XZ, and YY: Writing –review & editing. All authors contributed to the article and approved the submitted version.
