# Supplementary material for: Transcriptional regulation of TacL-mediated lipoteichoic acids biosynthesis by ComE during competence impacts pneumococcal transformation
Source: Front Cell Infect Microbiol. 2024 May 8;14:1375312. doi: 10.3389/fcimb.2024.1375312 (PMC11109429; doi:10.3389/fcimb.2024.1375312)
Supplement: Supplementary file 1 [file DataSheet_1.pdf]

Supplementary materials and data

S1 | Strains and plasmids used in this work

Named	Strains/plasmids	Relevant properties	Reference or source
	E. coli		
	DH5α	Cloning and plasmid maintenance strain	Sangon
	BL21	Protein expression strain	Sangon
	S. pneumoniae		
	D39	serotype 2, encapsulated	NCTC7466
	R6	non-encapsulated	ATCC BAA-255
	CPM8	Containing erythromycin ORF; Erm^R	(Lee and Morrison, 1999)
SPW1	D39Δ*tacL*	D39 derivative, the *tacL* region is replaced with *erm*; Erm^R	This work
SPW2	D39Δ*tacL::PJWtacL*	D39Δ*tacL::*pJWV25-*tacL*; SPW1 derivative, Zn^{2+}-dependent expression of TacL (P_{czcD}-*gfp*$^+$-*tacL*); Tet^R, Erm^R	This work
SPW3	D39Δ*cps*	D39Δ*dexB*-*cps2A*::JC derivative; the *dexB*-*cps2A* is deleted; Sr^R	(Zheng et al., 2017)
SPW4	D39Δ*cps*Δ*tacL*	SPW3 derivative, the *tacL* region is replaced with *erm*; Erm^R	This work
SPW5	D39Δ*cps*Δ*tacL::PJWtacL*	D39Δ*cps*Δ*tacL::*pJWV25-*tacL*; SPW4 derivative, Zn^{2+}-dependent expression of TacL (P_{czcD}-*gfp*$^+$-*tacL*); Tet^R, Erm^R	This work
SPW6	R6Δ*tacL*	R6 derivative, the *tacL* region is replaced with *erm*; Erm^R	This work
SPW7	R6Δ*tacL::PJWtacL*	R6Δ*tacL::*pJWV25-*tacL*; SPW6 derivative, Zn^{2+}-dependent expression of TacL (P_{czcD}-*gfp*$^+$-	This work

		tacL); Tet^R, Erm^R	
SPW8	D39s	D39 derivative, *rspL*^{K56T}; Kan^R	This work
SPW9	D39Δ*JHtacL*	SPW8 derivative, the JH*tacL* egion is replaced with JC; Sr^S, Kan^R	This work
SPW10	D39Δ*JH1*	SPW9 derivative; the JH1 region is mutated to the indicated sequence; Kan^S, Sr^R	This work
SPW11	D39Δ*JH2*	SPW9 derivative; the JH2 region is mutated to the indicated sequence; Kan^S, Sr^R	This work
SPW12	D39Δ*JH3*	SPW9 derivative; the JH3 region is mutated to the indicated sequence; Kan^S, Sr^R	This work
SPW13	D39Δ*comE*	D39 derivative, the *comE* region is replaced with *erm*; Erm^R	This work
SPW14	D39*::PJWcomE*^{WT}	D39::pJWV25*comE*^{WT}; D39 derivative, Zn^{2+}-dependent expression of ComE^{WT} (P_{czcD}-*gfp*$^+$-*comE*^{WT}); Tet^R	This work
SPW15	D39Δ*comE::PJWcomE*^{WT}	D39Δ*comE::*pJWV25-*comE*^{WT}; SPW14 derivative, the *comE* region is replaced with *erm*; Tet^R, Erm^R	This work
SPW16	D39*:: PJWcomE*^{D58E}	D39*::* pJWV25-*comE*^{D58E}; D39 derivative, Zn^{2+}-dependent expression of ComE^{D58E} (P_{czcD}-*gfp*$^+$-*comE*^{D58E}); Tet^R	This work
SPW17	D39Δ*comE::PJWcomE*^{D58E}	D39Δ*comE*::pJWV25-*comE*^{D58E}; SPW16 derivative, the *comE* region is replaced with *erm*; Tet^R, Erm^R	This work
SPW18	D39::pEVP3-*PcomE*	D39 derivative, *luc* under the control of *comE* promoter; Chl^R	This work
SPW19	D39Δ*comE*::pEVP3-*PcomE*	SPW18 derivative, the *comE* region is replaced with *erm*; Chl^R,	This work

		Erm^R	
SPW20	D39::pEVP3-*PtacL*	D39 derivative, *luc* under the control of *tacL* promoter; Chl^R	This work
SPW21	D39Δ*comE*::pEVP3-*PtacL*	SPW20 derivative, the *comE* region is replaced with *erm*; Chl^R, Erm^R	This work
SPW22	D39Δ*JD3*	SPW8 derivative, the JD3 region is replaced with JC; Sr^S, Kan^R	This work
SPW23	D39Δ*tacL*Δ*JD3*	SPW22 derivative, the *tacL* region is replaced with *erm*; Kan^R, Erm^R	This work
SPW24	D39Δ*tacL*Δ*cps*	SPW3 derivative, the *tacL* region is replaced with *erm*; Sr^R, Erm^R	This work
SPW25	D39Δ*JH2*Δ*JD3*	SPW11 derivative, the JD3 region is replaced with *erm*; Sr^R, Erm^R	This work
SPW26	D39Δ*JH2*Δ*cps*	SPW11 derivative, the *dexB-cps2A* region is replaced with *erm*; Sr^R, Erm^R	This work
	**Plasmid**		
	pIB166	*E. coli-S. pneumoniae* shuttle vector; Chl^R	(Biswas et al., 2008)
	pJWV25	N-terminal GFP fusions under control of a Zn^{2+} inducible promoter, integrates via double crossover at bga (P_{czcD}-*gfp*$^+$); Tet^R	(Eberhardt et al., 2009)
pWKF1	pJWV25-*tacL*	pJWV25 derivative, carrying *tacL* gene; Tet^R	This work
pWKF2	pJWV25-*comE*^{WT}	pJWV25 derivative, carrying *comE*^{WT} fragment; Tet^R	This work
pWKF3	pJWV25-*comE*^{D58E}	pJWV25 derivative, carrying *comE*^{D58E} fragment; Tet^R	This work
	pET-28a	protein expression vector; KanR	Takara
pWKF4	pET-28a-*comE*^{WT}	pET-28a derivative carrying the *comE*^{WT} orf fused to a (C-ter) His6 tag; KanR	This work

pWKF5	pET-28a-*comE*^{D58E}	pET-28a derivative carrying the *comE*^{D58E} orf fused to a (C-ter) His6 tag; KanR	This work
	pEVP3	*E. coli*-*S. pneumoniae* shuttle vector; Chl^{R}	(Pestova and Morrison, 1998)
pWKF6	pEVP3-*luc*	pEVP3 carrying the *luc* reporter gene; Chl^{R}	(Zhang et al., 2023)
pWKF7	pEVP3-P*tacL*-*luc*	pWKF6 derivative, *luc* under the control of *tacL* promoter; Chl^{R}	This work
pWKF8	pEVP3-*PcomE*-*luc*	pWKF6 derivative, *luc* under the control of *comE* promoter; Chl^{R}	This work

Abbreviations: Sr^{R} represents resistant to Streptomycin; Erm^{R} represents resistant to Erythromycin; Chl^{R} represents resistant to Chloramphenicol; Tet^{R} represents resistant to Tetracycline, and Kan^{R} represents resistant to Kanamycin.

References

Biswas, I., Jha, J. K.,Fromm, N. 2008. Shuttle expression plasmids for genetic studies in Streptococcus mutans. *Microbiology (Reading),* 154**,** 2275-2282.

Eberhardt, A., Wu, L. J., Errington, J., Vollmer, W.,Veening, J.-W. 2009. Cellular localization of choline-utilization proteins in *Streptococcus pneumoniae* using novel fluorescent reporter systems. *Molecular Microbiology,* 74**,** 395-408.

Lee, M. S.,Morrison, D. A. 1999. Identification of a new regulator in Streptococcus pneumoniae linking quorum sensing to competence for genetic transformation. *Journal of Bacteriology,* 181**,** 5004-5016.

Zhang, Y., Zhang, J., Xiao, J., Wang, H., Yang, R., Guo, X., et al. 2023. comCDE (Competence) operon is regulated by CcpA in *Streptococcus pneumoniae* D39. *Microbiology Spectrum,* 11**,** e0001223.

Zheng, Y., Zhang, X., Wang, X., Wang, L., Zhang, J.,Yin, Y. 2017. ComE, an essential response regulator, negatively regulates the expression of the capsular polysaccharide locus and attenuates the bacterial virulence in *Streptococcus pneumoniae*. *Frontiers in Microbiology,* 8**,** 277.

S2 | Primers used in this work

Name	Name Sequence (5′ – 3′)
*tacL*up-P1	AGACTACAGTGAAAATAGGAAATTT
*tacL*up-P2	ATCAAACAAATTTTGGGCCCGGGTTTTATAAGTTTGAAATCTTCTAC
*tacL*dw-P3	AATTCTATGAGTCGCTGCCGACTAATGAATCCTTTCTCTCCAAATCTG
*tacL*dw-P4	CTTTGCCCCAGGTATTCCTCAGGT
Erm-*F*	CCGGGCCCAAAATTTGTTTGAT
Erm-*R*	AGTCGGCAGCGACTCATAGAAT
tacL- F (SpeI)	GCTAAAGCTGGAACTAGTGGTTGAAATCAATAGGCTTTATTG
tacL-R (NotI)	CTCAGCTTATTATGCGGCCGCTTTAATCCGTCATGTCCGATAC
Pulldown-*tacL* F	biotin-AGCCTTGATATGGTGGATAAAATAG
Pulldown-*tacL* R	TAATTCCCAATAAAATCAGCTCTTT
FAM-*tacL* F	FAM-AGCCTTGATATGGTGGATAAAATAG
FAM-*tacL* R	TAATTCCCAATAAAATCAGCTCTTT
JH up-P1	GATTGGAGTAGTAGATGTCAAGG
JH up-P2	CAAGGAGTTTTCAGCATTATCCACCACTTTTTAGCAATTTG
JH dw-P3	CGTCCAAAAGCATAAGGAAAGAATCAGCAGATTTGGAGAGAA
JH dw-P4	GTCGTAAAGAGATAGATAATTGCTC
JC-F	GGATAATGCTGAAAACTCCTTGAAG
JC-R	CTTTCCTTATGCTTTTGGACGTTTAG
rspl P1	ATGCCTACAATTAACCAATTGG
rspl P2	TGAGTTAGGTTTTGTAGGTGTCATTGTTCCAACACGAGTTGC
rspl P3	TGACACCTACAAAACCTAACTCA
rspl P4	TTATGCTTTTGGACGTTTAGTAC
JH1-P2	CTGATTCAGATAATAGTAAGCTGCTTCGCATTGTACCACT
JH1-P3	AGTGGTACAATGCGAAGCAGCTTACTATTATCTGAATCAG
JH2-P2	CTGATTCAGATAATAGTGGATCATCCTATATTGTACCACT
JH2-P3	AGTGGTACAATATAGGATGATCCACTATTATCTGAATCAG
JH3-P2	CTGCTGATTTGAGCAATAGTAAGCTATCCTATATTGTACC
JH3-P3	GGTACAATATAGGATAGCTTACTATTGCTCAAATCAGCAG
comE up-P1	AACATGCTCATCACAAAAGA
comE up-P2	ATCAAACAAATTTTGGGCCCGGGATTGACAATTAGCAAGAA
comE dw-P3	ATTCTATGAGTCGCTGCCGACTTTAAAACTTTCATTCAAATTC
comE dw-P4	ACACAGATGAAATTGTTGGT
comE- F (SpeI)	CTAGCTAGCATGAAAGTTTTAATTTTAGAA
comE-R (NotI)	ATAAGAATGCGGCCGCTCACTTTTGAGATTTTTTCTC
comE-His F	GAGATTTTTGGTCATTAATGGTGATGGTGATGGTGCTTTTGAGATTTTTTCTCTA
comE-His R	CCGCTCGAGATTTTTGGTCATTATTTATCATCATCATCT
comE^{D58E}-P2	CCATGAATATCGAT C TCTAGGAAATAAAGC
comE^{D58E}-P3	GCTTTATTTCCTAGAG ATCGATATTCATGG
gfp-*F*	AAAGGAGAAGAACTTTTCACTGGAG
gfp-*R*	AGTAGTGACAAGTGTTGGCCATGGA

comE-luc-F	CCGCTCGAGCTAGTTCTTGTTGAACAAA
comE-luc-R	AAGGCCTGGATCCTTCAAAGCTACAAACTGTTCC
tacL-luc-F	AGCTTATCGATACCGTCGACCTCGAGGCAAAATCAGACTTATCGGG
tacL-luc-R	GTAAAGAGCTGATTTTATTGGATCCAGGAGGAATAATGAGATCCGC
JD3-P1	CCATAATAATAACCGATGGTGTG
JD3-P2	AGGAGTTTTCAGCATTATCCAATTGATCAGGACAGTCAAA
JD3-P3	GTCCAAAAGCATAAGGAAAGGGTTCGCGGGAAGTCTACTAA
JD3-P4	CATAGGTGTCAATTCCACTAACATA
dexB-*cps2A*-P1	GAGGTCGTTCATGAGCAACTC
dexB-*cps2A*-P4	GACTGAATCGTGTCATAAGTCAC
gyrB-F	GTTCGTATGCGTCCAGGGAT
gyrB-R	ATACCACGCCCATCATCCAC
tacL-F	AGCGAACAGGAGCAACAGAA
tacL-R	ATGCTGGATGGCCTTGTTT
comE-F	TCGTCATTACAATCCTTACGCTA
comE-R	ACCCCTGTTGTTTCAATATACAAA

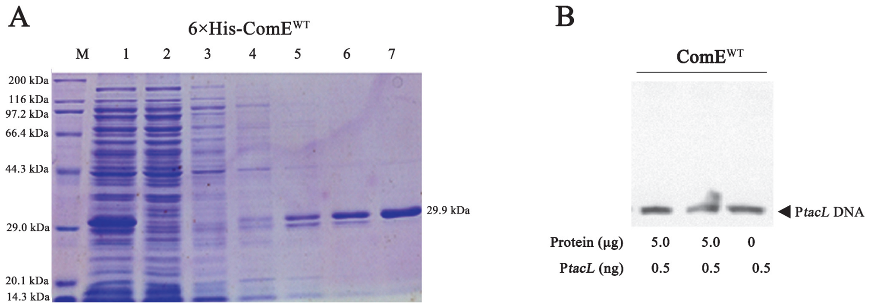

Fig. S1 Wild-type ComE did not bind to the upstream regulatory region of *tacL*

(A) The recombinant 6×His-ComE^{WT} protein were analyzed by SDS-PAGE and migrated with an apparent molecular mass of 29.9 kDa. Lane M: Marker; Lane 1, Protein expression induced by IPTG; Lane 2: Ni-NTA flow-through; Lanes 3 – 6: elution from the columns with imidazole at 20, 40, 80 and 500 mM, respectively. Lane 7: the purified form of 6×His-ComE^{WT}.

(B) EMSA results of binding between ComE^{WT} and P*tacL*. ComE^{WT} could not bind to the P*tacL*.

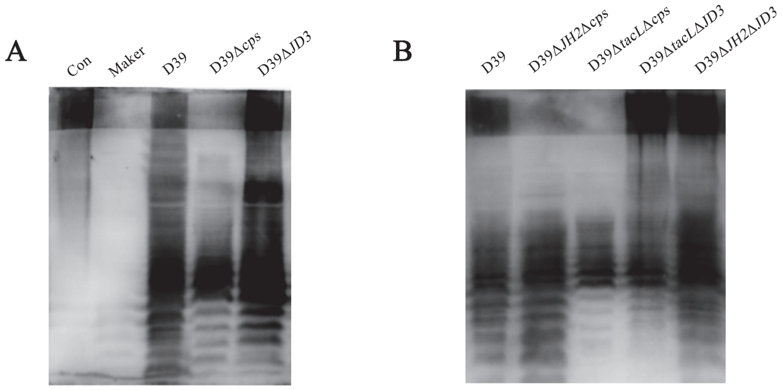

Fig. S2 The amount of CPS in pneumococcal strains with different genotypes

(A) The amount of CPS in the indicated strains determined by Western blot.

(B) The levels of CPS in the indicated strians was determined by Western blot.
